# Research on Delamination Damage Factor of Hole-Making Process Optimization Based on Carbon Fiber Composite Materials

**DOI:** 10.3390/polym18020219

**Published:** 2026-01-14

**Authors:** Linsheng Liu, Yushu Lai, Yiwei Zhang, Lin Huang, Jiexiao Yang, Yuchi Jiang, Zhiwei Hu, Zhen Li, Bin Wang

**Affiliations:** 1 School of Mechanical Engineering, Chongqing Three Gorges University, Chongqing 404100, China; 17623257368@163.com (L.L.);; 2 Intelligent Manufacturing Industry Technology Research Institute, Sichuan University of Arts and Science, Dazhou 635000, China; 3 Chongqing Jiangdong Machinery Co., Ltd., Chongqing 404032, China; 4 School of Mechatronic Engineering, Southwest Petroleum University, Chengdu 610500, China

**Keywords:** CFRP, delamination damage factor, drilling, variable parameters, hole making process

## Abstract

Carbon fiber reinforced polymer (CFRP) is prone to delamination damage during drilling, which seriously affects the processing quality. This study focuses on the use of variable parameter drilling technology. Firstly, an anisotropic constitutive model and a Hashin failure model for CFRP were constructed. Then, based on ABAQUS and VUMAT user subroutines, the influence laws of cutting parameters (spindle speed and feed rate) on delamination damage were explored. For the two methods of conventional fixed parameter drilling and variable parameter drilling (dynamic adjustment of feed rate when the drill reaches the exit plane), comparative simulation experiments were conducted. Subsequently, the genetic algorithm was introduced to optimize the spindle speed and feed rate under the variable parameter mode, and the results were verified through hole-making experiments. The results show that: under a constant spindle speed, the delamination damage factor increases monotonically with the increase in feed rate; under a constant feed rate, the delamination damage factor decreases first and then increases with the increase in spindle speed, presenting a nonlinear change characteristic. Among them, the variable parameter strategy of “first high feed, then low feed” can significantly reduce the delamination damage; the obtained optimal parameters can effectively balance the drilling quality and processing efficiency. This research provides theoretical and experimental support for optimizing CFRP hole-making parameters, addressing delamination control challenges in traditional drilling, and facilitating CFRP applications in aerospace and intelligent manufacturing.

## 1. Introduction

Carbon fiber-reinforced polymers (CFRP) have been widely used in the aerospace and medical device industries due to their outstanding qualities [1,2]. Drilling is a crucial step in the machining of composite materials because of CFRPs’ exceptional wear resistance and ultra-high hardness [3,4]. Delamination, ripping, and burrs are examples of machining damage that can seriously reduce the overall performance and service life of CFRP components during the drilling process [5,6,7,8,9,10,11]. Defects include hole wall damage, heat damage, and dimensional geometric errors that can occur in addition to typical drilling damage [12,13,14]. Among various types of damage, delamination damage has the most significant impact on the performance of CFRP components. The delamination damage zone expands as the drill bit wears and cutting temperatures rise. This occurs because excessive heat softens the substrate, weakening interlaminar bonding strength and thereby enlarging the delamination damage zone [15,16]. As a result, reducing delamination damage during CFRP drilling has emerged as a crucial technological issue.

Researchers from all over the world have carried out in-depth studies to lessen the harm caused by drilling CFRP. According to an experimental study, the degree of delamination and surface quality are directly influenced by the choice of machining parameters and the drill bit’s geometric structure [17,18,19,20]. Researchers conducted machining and drilling experiments using drills made of different materials and featuring various structures, achieving certain research results. However, due to issues concerning machining costs and efficiency, large-scale mass production remains unfeasible [21,22]. Therefore, it is necessary to conduct in-depth research on conventional drilling processes. Krishnarao et al. [23] used carbide drill bits to penetrate carbon fiber reinforced composite laminates. Through a series of orthogonal experiments, they conducted variance analysis on several factors, including drilling force, hole diameter, entry delamination, and exit delamination. Ultimately, the optimized cutting parameters were determined to be a feed rate of 0.137 mm/rev and a spindle speed of 12,000 rpm. Zhan et al. [24,25,26] conducted drilling experiments on carbon fiber reinforced composites using carbide twist drills. They investigated the influence of machining parameters on the quality of the drilled holes. Experimental results indicate that variations in feed rate are the primary factor causing changes in axial force, which in turn is the main cause of delamination damage. Under consistent tool conditions, increasing the feed rate and reducing rotational speed will increase axial force during drilling, thereby exacerbating delamination damage. Furthermore, setting the feed rate too high will lead to increased axial force during drilling, which not only intensifies interlayer damage but also results in increased surface roughness [15,27].

**Figure 1:**
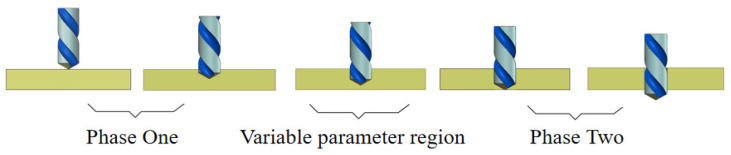
Variable parameter hole-making process.

Overall, while previous research has elucidated the relationship between cutting parameters and delamination damage and carried out thorough investigations into drill bit structural optimization and machining process enhancements, these research efforts have been predicated on fixed machining parameters and have not yet explored the viability of dynamic parameter control. For conventional twist drills, an effective delamination control scheme that comprehensively balances machining quality and efficiency remains elusive. Particularly concerning the timing of parameter switching and optimization strategies during “variable parameter” processes, systematic conclusions have yet to be established. Therefore, to further overcome the bottleneck in controlling delamination damage, it is necessary to explore variable-parameter drilling processes for CFRP, thereby providing new avenues for optimizing CFRP drilling techniques. The variable parameter drilling method involves cutting to a specified depth followed by either retraction or non-retraction operations [16]. This paper describes advancing the drill to a certain depth, altering machining parameters, and then completing the drilling process. Figure 1 illustrates the workflow for “variable parameter” drilling.

This study employs ABAQUS 2021 software to simulate the drilling process of CFRP, aiming to provide a theoretical basis and technical reference for suppressing delamination damage during the drilling of this material. The simulation results not only validated the influence patterns of spindle speed and feed rate on delamination damage but also confirmed that variable-parameter drilling processes can effectively reduce the occurrence of delamination damage. Finally, genetic algorithms were employed to optimize the variable-parameter drilling process, yielding the optimal combination of process parameters. This research approach not only addresses the shortcomings of existing studies but also provides a novel solution for optimizing CFRP drilling process parameters, offering both theoretical and practical value.

## 2. Establishment of CFRP Drilling Model

### 2.1. The Constitutive Model of CFRP Material

When creating its constitutive model, fiber orientation is usually reduced to an anisotropic material form due to its anisotropic characteristics, which lowers the number of independent elastic constants needed for analysis. Their stress–strain relationship is represented by the stiffness matrix of anisotropic elastic materials, as shown in Equations (1) and (2) [28]:

(1) σ=Cε

(2) $$\left[\begin{array}{c} \sigma_{1}\\ \sigma_{2}\\ \sigma_{3}\\ \sigma_{4}\\ \sigma_{5}\\ \sigma_{6} \end{array}\right]=\left[\begin{array}{cccccc} C_{11} & C_{12} & C_{13} & C_{14} & C_{15} & C_{16}\\ C_{21} & C_{22} & C_{22} & C_{24} & C_{25} & C_{26}\\ C_{31} & C_{32} & C_{33} & C_{34} & C_{35} & C_{36}\\ C_{41} & C_{42} & C_{43} & C_{44} & C_{45} & C_{46}\\ C_{51} & C_{52} & C_{53} & C_{54} & C_{55} & C_{56}\\ C_{61} & C_{62} & C_{63} & C_{64} & C_{65} & C_{66} \end{array}\right]\left[\begin{array}{c} \varepsilon_{1}\\ \varepsilon_{2}\\ \varepsilon_{3}\\ \varepsilon_{4}\\ \varepsilon_{5}\\ \varepsilon_{6} \end{array}\right]$$

or the purpose of simplifying the calculations, the CFRP is modeled as an orthotropic anisotropic material. This material exhibits three mutually perpendicular elastic symmetry planes, resulting in a stiffness coefficient that is equal to zero. This relationship is illustrated in Equation (3):

(3) $$C_{14}=C_{16}=C_{24}=C_{26}=C_{34}=C_{36}=C_{45}=C_{56}=0$$

The stress–strain relationship of the orthotropic anisotropic material CFRP is shown in Equations (4) and (8):

(4) $$\left[\begin{array}{c} \sigma_{1}\\ \sigma_{2}\\ \sigma_{3}\\ \sigma_{4}\\ \sigma_{5}\\ \sigma_{6} \end{array}\right]=\left[\begin{array}{cccccc} C_{11} & C_{12} & C_{13} & 0 & 0 & 0\\ C_{21} & C_{22} & C_{23} & 0 & 0 & 0\\ C_{31} & C_{32} & C_{33} & 0 & 0 & 0\\ 0 & 0 & 0 & C_{44} & 0 & 0\\ 0 & 0 & 0 & 0 & C_{55} & 0\\ 0 & 0 & 0 & 0 & 0 & C_{66} \end{array}\right]\left[\begin{array}{c} \varepsilon_{1}\\ \varepsilon_{2}\\ \varepsilon_{3}\\ \varepsilon_{4}\\ \varepsilon_{5}\\ \varepsilon_{6} \end{array}\right]$$

(5) $$\begin{array}{c} \begin{array}{c} C_{11}=E_{1}\left(1-v_{23}v_{32}\right)\gamma ,C_{12}=E_{1}\left(v_{21}+v_{31}v_{23}\right)\gamma =E_{2}\left(v_{12}+v_{13}v_{32}\right)\gamma ,C_{44}=G_{12} \end{array} \end{array}$$

(6) $$\begin{array}{c} C_{22}=E_{2}\left(1-v_{13}v_{31}\right)\gamma ,C_{13}=E_{1}\left(v_{31}+v_{21}v_{32}\right)\gamma =E_{3}\left(v_{13}+v_{12}v_{23}\right)\gamma ,C_{55}=G_{13} \end{array}$$

(7) $$\begin{array}{c} C_{33}=E_{3}\left(1-v_{12}v_{21}\right)\gamma ,C_{23}=E_{1}\left(v_{32}+v_{12}v_{31}\right)\gamma =E_{3}\left(v_{23}+v_{21}v_{13}\right)\gamma ,C_{66}=G_{23} \end{array}$$

(8) $$\gamma =\frac{1}{1-v_{12}v_{21}-v_{23}v_{32}-v_{13}v_{31}-2v_{21}v_{13}v_{32}}$$

In the formula, $\sigma_{1}$, $\sigma_{21}$, and $\sigma_{3}$ represent the normal stress components; $\sigma_{4}$, $\sigma_{5}$, and $\sigma_{6}$ represent the shear stress components. Furthermore, $\sigma_{1}$ = $\sigma_{11}$, $\sigma_{2}$ = $\sigma_{22}$, and $\sigma_{3}$ = $\sigma_{33}$.

In engineering, elastic constants are commonly used to characterize the elastic properties of materials. The stress–strain relationship for CFRP orthotropic materials is expressed as shown in Equation (9):

(9) $$\left[\begin{array}{c} \varepsilon_{11}\\ \varepsilon_{22}\\ \varepsilon_{33}\\ \gamma_{12}\\ \gamma_{13}\\ \gamma_{23} \end{array}\right]=\left[\begin{array}{cccccc} \frac{1}{E_{1}} & \frac{-v_{21}}{E_{2}} & \frac{-v_{31}}{E_{3}} & 0 & 0 & 0\\ \frac{-v_{12}}{E_{1}} & \frac{-v_{12}}{E_{1}} & \frac{-v_{32}}{E_{3}} & 0 & 0 & 0\\ \frac{-v_{13}}{E_{1}} & \frac{-v_{23}}{E_{2}} & \frac{1}{E_{3}} & 0 & 0 & 0\\ 0 & 0 & 0 & \frac{1}{G_{12}} & 0 & 0\\ 0 & 0 & 0 & 0 & \frac{1}{G_{13}} & 0\\ 0 & 0 & 0 & 0 & 0 & \frac{1}{G_{23}} \end{array}\right]\left[\begin{array}{c} \varepsilon_{11}\\ \varepsilon_{22}\\ \varepsilon_{33}\\ \tau_{12}\\ \tau_{13}\\ \tau_{23} \end{array}\right]$$

In the formula, $\varepsilon_{ii}$ denotes normal strain; $\gamma_{ij}$ signifies shear strain; $\sigma_{ii}$ indicates normal stress; $\tau_{ij}$ represents shear stress; *E_i_* refers to elastic modulus; $G_{ij}$ corresponds to shear modulus; and $V_{ij}$ stands for Poisson’s ratio.

### 2.2. Refinement of the Failure Model for CFRP Materials

Expressions for Hashin failure modes [29] are given in Equations (10)–(13):

① Fiber tensile failure ($\sigma_{11}\geq 0$):

(10) $$F_{ft}={\left(\frac{\sigma_{11}}{X_{T}}\right)}^{2}+{\left(\frac{\tau_{12}}{S_{12}}\right)}^{2}+{\left(\frac{\tau_{13}}{S_{13}}\right)}^{2}$$

② Fiber compression failure ($\sigma_{11}<0$):

(11) $$F_{fc}={\left(\frac{\sigma_{11}}{X_{C}}\right)}^{2}$$

③ Failure of the matrix under tensile stress ($\sigma_{22}+\sigma_{33}\geq 0$):

(12) $$F_{mt}={\left(\frac{\sigma_{22}+\sigma_{33}}{Y_{T}}\right)}^{2}+{\left(\frac{\tau_{12}}{S_{23}}\right)}^{2}-\left(\frac{\sigma_{22}\times \sigma_{33}}{{S_{23}}^{2}}\right)+\left(\frac{{\tau_{12}}^{2}-{\tau_{13}}^{2}}{{S_{13}}^{2}}\right)$$

④ Compressive failure of the matrix ($\sigma_{22}+\sigma_{33}<0$):

(13) $$F_{mc}=\frac{\sigma_{22}\times \sigma_{33}}{4\times {S_{23}}^{2}}-\frac{\sigma_{22}+\sigma_{33}}{Y_{C}}+{\left(\frac{\sigma_{22}+\sigma_{33}}{2\times S_{23}}\right)}^{2}+\frac{\tau_{23}^{2}-\left(\sigma_{22}\times \sigma_{33}\right)}{{S_{23}}^{2}}+\frac{{\tau_{12}}^{2}+{\tau_{13}}^{2}}{{S_{12}}^{2}}$$

In the formula, $\sigma_{ii}$ and $\tau_{ij}$ denote the normal stress and shear stress, respectively. $X_{T}$ and $Y_{T}$ represent the tensile strength along the fiber direction and perpendicular to it. $X_{C}$ and $Y_{C}$ indicate the compressive strength along the fiber direction and in a direction perpendicular to it. $S_{ij}$ refers to the shear strength within the plane.

When the integral points satisfy the initial conditions for failure, the material will undergo stiffness degradation. The evolution of the stiffness degradation coefficient adheres to an exponential damage evolution law. The four damage factors corresponding to the four failure conditions are presented in Table 1. The calculation formula for the deformed stiffness matrix is shown in Equations (14)–(16):

(14) $$\left[\begin{array}{c} \sigma_{1}\\ \sigma_{2}\\ \sigma_{3} \end{array}\right]=\left[\begin{array}{ccc} \left(1-d_{f}\right)C_{11} & \left(1-d_{f}\right)\left(1-d_{m}\right)C_{12} & \left(1-d_{f}\right)C_{12}\\ 0 & \left(1-d_{m}\right)C_{22} & \left(1-d_{m}\right)C_{23}\\ 0 & 0 & C_{33} \end{array}\right]\left[\begin{array}{c} \varepsilon_{1}\\ \varepsilon_{2}\\ \varepsilon_{3} \end{array}\right]$$

(15) $$\begin{array}{c} \begin{array}{c} d_{f}=\left\{\begin{array}{c} \begin{array}{cc} d_{ft} & \sigma_{1}\geq 0 \end{array}\\ \begin{array}{cc} d_{fc} & \sigma_{1}<0 \end{array} \end{array}\right. \end{array} \end{array}$$

(16) $$\begin{array}{c} \begin{array}{c} d_{m}=\left\{\begin{array}{c} \begin{array}{cc} d_{mt} & \sigma_{2}+\sigma_{3}\geq 0 \end{array}\\ \begin{array}{cc} d_{mc} & \sigma_{2}+\sigma_{3}<0 \end{array} \end{array}\right. \end{array} \end{array}$$

Among these, $G_{ft}$ and $G_{fc}$ denote the tensile and compressive fracture energies of the fibers, respectively; $G_{mc}$ and $G_{mt}$ represent the tensile and compressive fracture energies of the matrix. Additionally, $\varepsilon_{it}^{f}$ and $\varepsilon_{ic}^{f}$ denote the components of the strain tensor.

In this study, the VUMAT subroutine was used to substitute the user-defined material parameters into the initial failure criterion of the material and the calculation formula for the damage factor.

### 2.3. Constitutive Model for Cohesive Material

The debonding behavior of delamination defects in structures is usually modeled in finite element simulations using cohesive elements based on fracture mechanics. Cohesive elements based on element properties are used in this study to quantitatively analyze delamination defects and fully characterize the delamination behavior during the hole-making process. To characterize the delamination behavior during the hole-making process and quantitatively analyze the delamination damage, cohesive elements based on element properties are employed in this study. The Cohesive element employs the tensile-separation criterion of fracture mechanics, with its stress–strain relationship given by Equation (17) [30,31].

(17) $$\sigma =\left[\begin{array}{c} \sigma_{n}\\ \sigma_{s}\\ \sigma_{t} \end{array}\right]=\left[\begin{array}{ccc} E_{nn} & & \\ & E_{ss} & \\ & & E_{tt} \end{array}\right]\left[\begin{array}{c} \varepsilon_{n}\\ \varepsilon_{s}\\ \varepsilon_{t} \end{array}\right]=\left[\begin{array}{ccc} K_{nn} & & \\ & K_{ss} & \\ & & K_{tt} \end{array}\right]\left[\begin{array}{c} \delta_{n}\\ \delta_{s}\\ \delta_{t} \end{array}\right]$$

Among these, $\sigma_{n}$, $\sigma_{s}$, and $\sigma_{t}$ represent the tensile stresses at cracking; $E_{nn}$, $E_{ss}$, and $E_{tt}$ denote the material elastic moduli; $K_{nn}$, $K_{ss}$, and $K_{tt}$ indicate the material stiffnesses; $\varepsilon_{n}$, $\varepsilon_{s}$, and $\varepsilon_{t}$ signify the strains; and $\delta_{n}$, $\delta_{s}$, and $\delta_{t}$ denote the opening displacements after damage initiation.

To enhance simulation accuracy, a secondary stress criterion is adopted as the damage initiation criterion, as shown in Equation (18):

(18) $${\left(\frac{\sigma_{n}}{N_{max}}\right)}^{2}+{\left(\frac{\sigma_{s}}{S_{max}}\right)}^{2}+{\left(\frac{\sigma_{t}}{T_{max}}\right)}^{2}=1$$

Damage evolution of cohesive unit in bonding layer material. The stress update after damage initiation is shown in Equations (19)–(21):

(19) $$\sigma =\left\{\begin{array}{c} (1-D){\bar{\sigma }}_{n}\;\;\;\;\;\;\;\;\;\;\;\;\;{\bar{\sigma }}_{n}\geq 0\\ \;\;{\bar{\sigma }}_{n}\;\;\;\;\;\;\;\;\;\;\;\;\;\;\;\;\;\;\;\;\;\;\;\;\;\;\bar{\sigma_{n}}<0 \end{array}\right.$$

(20) $$\sigma_{s}=(1-D){\bar{\sigma }}_{s}$$

(21) $$\sigma_{t}=(1-D){\bar{\sigma }}_{t}$$

Among these, D represents the damage state variable.

This study employs an energy-based composite model comprising Type I, Type II, and Type III fracture mechanisms. Material parameters are listed in Table 2 [31]. *G_n_*, *G_s_*, and *G_t_* denote the critical fracture energies.

### 2.4. Cutting Tools, Workpiece Models

**Figure 2:**
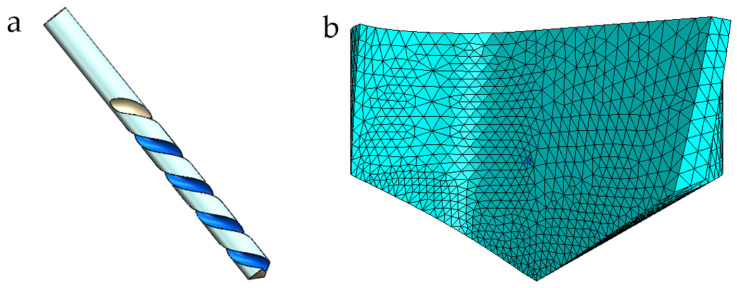
Complete Tool: (**a**) twist drill bit; (**b**) mesh division of the drill bit.

A typical twist drill is used in this tool model. The tool was loaded into ABAQUS after being modeled using SolidWorks 3D 2022 software, as seen in Figure 2a. Only the part of the tool used for drilling was chosen and meshed in order to minimize the number of mesh elements and save computational time. Figure 2b shows the particular tool geometry and mesh setup with C3D4 mesh attributes. During simulation, tool wear and distortion were disregarded. All tool mesh elements were connected to a reference point (RP) by “rigid body coupling.” The associated RP was subjected to further tool loads. The drill bit was composed of cemented carbide and had a diameter of 10 mm, a point angle of 120 degrees, and a helix angle of 30 degrees. Table 3 is a detailed description of the tool’s physical characteristics.

**Figure 3:**
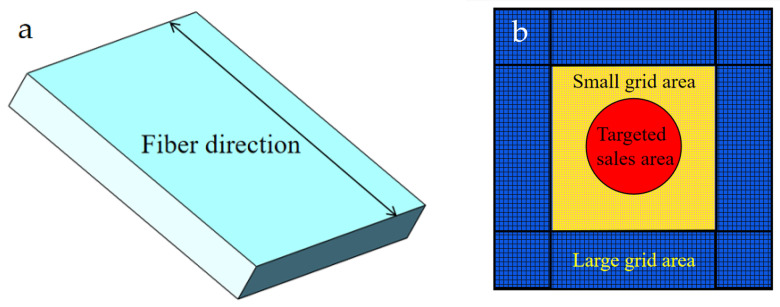
Workpiece model: (**a**) macro model; (**b**) grid division area.

The workpiece model’s overall measurements are 30 mm by 30 mm by 4 mm. There are twenty layers of carbon fiber, each with a thickness of 0.2 mm. The unidirectional carbon fiber reinforced composite material is defined as an equivalent homogeneous model. A three-dimensional macro model of the drilling process is established and meshed, as shown in Figure 3. Grid type selection and partitioning quality directly impact model convergence speed and simulation accuracy. To ensure mesh quality in the drill contact area, the machined region and unmachined regions of the workpiece were treated separately. The workpiece mesh properties were set to C3D8R.

The material property parameters required for establishing the workpiece model are shown in Table 4 [32,33].

### 2.5. Boundary Conditions and Load Settings

This paper employs contact pairs with higher solution efficiency to define the contact relationships within the model. When setting up contacts, the “Surface to Surface” mode in ABAQUS is utilized, which represents a face-to-face contact discretization method. The specific contact surface to be defined is the interface between the drill bit and the surface of the carbon fiber reinforced composite plate. Within this model, the mesh density at the same contact location is higher for the carbon fiber reinforced composite plate than for the drill bit. In contact analysis, the normal characteristics are defined by a hard contact model, and the tangential friction properties between contact surfaces are simulated using a penalty friction model. According to experimental findings in pertinent literature, the friction coefficient between the drill bit and the carbon fiber-reinforced composite material is 0.15 [30]. At the same time, to ensure the convergence of computational results, it is necessary to apply small loads or small displacement boundary conditions to the contact surfaces when contact occurs, thereby ensuring the smooth establishment of contact relationships between all surfaces. Limit all degrees of freedom on the workpiece’s peripheral surfaces by fully clamping them. Set the software’s constraint loads to U1 = U2 = U3 = UR1 = UR2 = UR3 = 0. The drill bit’s x, y, and angular displacement are all fixed. The software load constraints are set to U1 = U2 = UR1 = UR2 = 0. A velocity load $f_{v}$ is applied to the drill bit in the z direction, as well as an angular velocity load about the z axis. Defining the drill bit as a rigid body can reduce simulation time without compromising computational accuracy. Its motion is achieved by applying velocity and displacement to a reference point.

## 3. Results and Discussion

### 3.1. Verification of CFRP Drilling Simulation Model

In order to better evaluate the quality of the hole-making process, this paper uses the delamination damage factor to assess the processing results. Three types of quantitative evaluation metrics are used in the assessment process for CFRP delamination damage: one-dimensional diameter evaluation, two-dimensional area evaluation, and three-dimensional volume evaluation. In this paper, a quantitative analysis is conducted using the area-based stratified defect factor, among which the stratified defect factor is $F_{A}=\frac{A_{max}}{A_{norm}}$.

To further evaluate the effect of spindle drilling speed on hole machining quality, drilling operations were performed on CFRP material using machining scheme 1 (details in Table 5).

**Figure 4:**
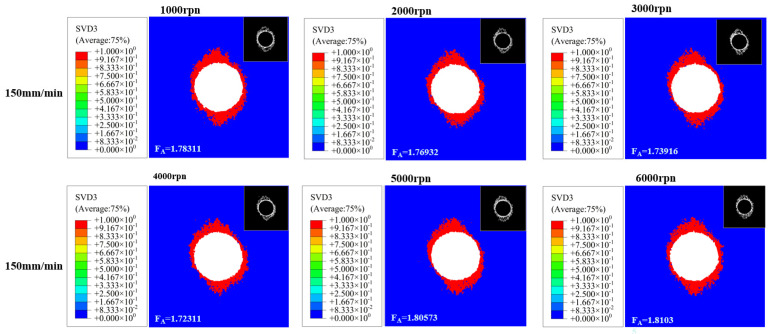
Simulation results of drilling for Scheme 1.

Drilling results under different machining parameters were obtained through Abaqus simulation. The delamination factor was extracted from the characteristic boundary using the Sobel algorithm in MATLAB R2019a. Subsequently, the two-dimensional delaminated area was calculated using Fiji ImageJ software, yielding the delamination damage factor results as shown in Figure 4.

To further evaluate the impact of feed rate on drilling quality, drilling operations were performed on CFRP material using Machining Scheme 2 (details in Table 6).

**Figure 5:**
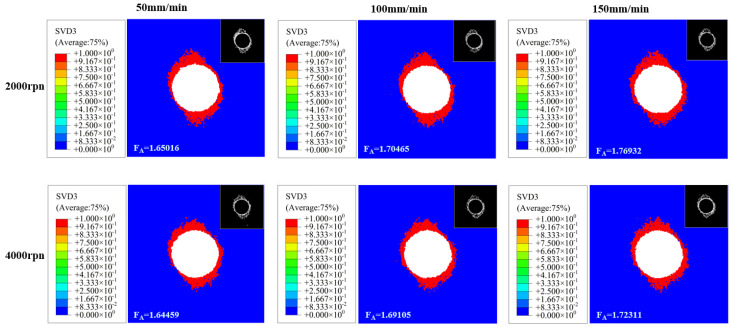
Simulation results of drilling for Scheme 2.

Drilling results under different machining parameters were obtained through Abaqus simulation. The delamination factor was extracted from the characteristic boundary using the Sobel algorithm in MATLAB. Subsequently, the two-dimensional delaminated area was calculated using ImageJ software, yielding the delamination damage factor results as shown in Figure 5.

By conducting ABAQUS simulations combined with processing schemes 1 and 2, the influence of cutting parameters on the delamination damage of CFRP was first clarified. When the feed rate is fixed at 150 mm/min, the delamination damage factor ($F_{A}$) shows a trend of “first decreasing and then increasing” as the spindle speed increases. Before the critical rotational speed (approximately 4000 rpm), the increase in the spindle speed leads to an increase in the number of contacts between the tool and the material, and the axial force per cut becomes more dispersed. The $F_{A}$ gradually decreases. When exceeding the critical rotational speed, chip morphology at high speeds may become more irregular or difficult to evacuate smoothly. Chip accumulation within the hole or tool flutes may induce additional squeezing and friction against the hole wall, introducing extra radial forces or torque. This promotes interlaminar separation expansion, weakening the bond strength at fiber-matrix interfaces. Fibers remain susceptible to “pull-out” phenomena, and $F_{A}$ increases linearly with spindle speed (e.g., $F_{A}$ at 6000 rpm is significantly higher than at 4000 rpm). When the spindle speed is fixed 2000 rpm or 4000 rpm), $F_{A}$ increases monotonically as the feed rate increases. For example, when the spindle speed is 4000 rpm, the feed rate increases from 50 mm/min to 150 mm/min. The $F_{A}$ value rises from 1.64459 to 1.72311. This is because the increase in feed rate leads to a concentrated cutting load per single cut, resulting in a significant increase in the axial force of drilling, which directly aggravates the interlayer separation.

### 3.2. Research on Variable Parameter Drilling Process of CFRP

**Figure 6:**
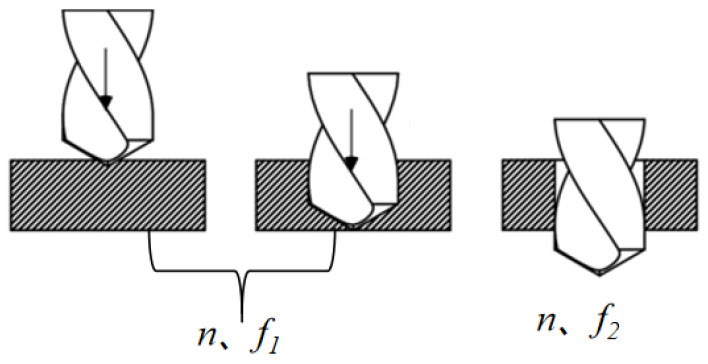
Schematic diagram of drill bit position when parameters change. The shaded area represents CFRP panels, and the black arrow indicates the downward feed rate.

The quality of the machining process is greatly impacted by the machining parameter modification locations when using CFRP variable-parameter drilling. Consequently, it is essential to identify these variable parameter positions prior to initiating the variable parameter operations [16]. In this study, we have opted to modify the feed rate upon the drill tip reaching the exit plane. The corresponding positional diagram is illustrated in Figure 6. When processing a single-diameter hole, feed rate (*f*) is a key factor affecting the quality of hole processing. Therefore, the variable parameter adopted in this paper refers to the variable feed rate.

To validate the effectiveness of variable-parameter drilling, an experimental design was established comparing conventional drilling (Scheme 3) with variable-parameter drilling (Scheme 4), as detailed in Table 7. Scheme 3 employed fixed feed rates and spindle speeds throughout the entire drilling process. Scheme 4 utilized the same machining parameters as Scheme 1 during the initial stage, then switched to variable-parameter conditions at a predetermined position. Subsequent hole sections were machined by adjusting the feed rate, enabling a comparison of the performance differences between the two processes.

**Figure 7:**
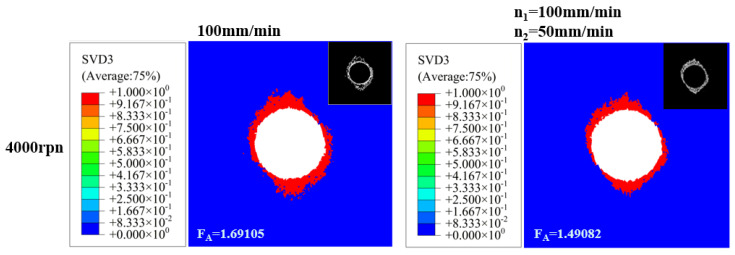
Conventional hole-making and variable-parameter hole-making.

The outlet delamination damage resulting from both conventional hole-making and variable-parameter hole-making methods is illustrated in Figure 7.

Comparing Scheme 3 with Scheme 4 reveals that Scheme 3 exhibits severe tearing and delamination at the exit, with *F_A_* reaching 1.69105. In contrast, Scheme 4 reduced the feed rate from $f_{1}$ = 100 mm/min to $f_{2}$ = 50 mm/min upon reaching the exit plane, significantly decreasing delamination damage. The *F_A_* decreased to 1.49082, representing an 11.8% reduction compared to Scheme 3. Due to variations in grid refinement levels, differences in RP selection, and measurement errors, the simulation results exhibit a certain degree of experimental scatter at 11.8%. Subsequent testing on a machining center confirmed that fiber breakage and resin delamination at the exit were markedly reduced in Scheme 4 compared to Scheme 3, demonstrating that the “low feed rate at the exit section” strategy effectively suppresses delamination damage.

### 3.3. Optimization of Drilling Parameters

In the optimization of the drilling process parameters for CFRP, the optimization objective is the maximum material removal rate (the drilling depth ($\alpha_{p}$) is half of the drill bit diameter, and the optimization objective function is shown in Equation (22)). The decision variables are the spindle speed and feed rate; in terms of constraints, the quality assessment index is the layer defect, and the maximum layer factor is 1.6 (based on GB/T 5783-2000 [34], the maximum inner diameter of M10 coarse thread full-thread hexagonal head bolt is 16 mm). This document adopts the standard based on its methodological value as a conservative reference benchmark for quantifying process improvements, rather than as an acceptance criterion for aerospace CFRP structures. When the delamination size exceeds the size of the screw cap, it is considered that the processing quality of the hole does not meet the quality requirements. Thus, the maximum delamination factor at the outlet side of the drill can be determined as 1.6. The calculation formula for the delamination factor is shown in Equation (23) [35]. The spindle speed range is 1000~6000 rpm and the feed rate follows the strategy of “high feed first, then low feed” (the constraints are shown in Equation (24)).

(22) $$\frac{1}{Q}=\frac{1}{\pi \alpha_{p}^{2}nf_{1}}+\frac{1}{\pi \alpha_{p}^{2}nf_{2}}$$

(23) $$F_{A}=1.78n^{-0.7991}\times f^{0.623}+1.115\leq 1.6$$

(24) $$\begin{array}{c} min\frac{1}{Q}=\frac{1}{\pi \alpha_{p}^{2}nf_{1}}+\frac{1}{\pi \alpha_{p}^{2}nf_{2}}\\ s.t\left\{\begin{array}{c} \begin{array}{c} \begin{array}{c} F_{A}=1.78n^{-0.7991}\times {f_{1}}^{0.623}+1.115\leq 1.6\\ F_{A}=1.78n^{-0.7991}\times {f_{2}}^{0.623}+1.115\leq 1.6 \end{array}\\ 1000\;\mathrm{rpm}\leq n\leq 6000\;\mathrm{rpm} \end{array}\\ \begin{array}{c} \begin{array}{c} 50\;\mathrm{mm}/\min\ll f_{1}\leq 225\;\mathrm{mm}/\min\\ 25\;\mathrm{mm}/\min\ll f_{2}\leq 225\;\mathrm{mm}/\min \end{array}\\ f_{2}<f_{1} \end{array} \end{array}\right. \end{array}$$

Genetic algorithm is a global search algorithm derived by imitating the natural selection and genetic process of living organisms [36]. Compared with traditional exact algorithms, it can handle more complex situations. In this paper, considering the specific circumstances and actual requirements, the traditional genetic algorithm has been reasonably improved. In the genetic algorithm, the population size is set to 100, the crossover rate is 0.8, and the mutation rate is 0.2. The objective function and constraint conditions to be optimized were input into the mathematical software. After 100 iterations, the optimal parameter combination was obtained: spindle speed of 3598 r/min, feed rate for the front section $f_{1}=132\;mm/min$ and feed rate for the rear section $f_{2}=92\;mm/min$.

Under optimal parameter conditions ($f_{1}=132\;mm/min$, depth 4 mm;${\;f}_{2}=92\;mm/min$, depth 3 mm), the processing time per hole is only 3.77 s. Compared to the initial variable parameter scheme ($f_{1}=100\;mm/min$, depth 4 mm; ${\;f}_{2}=20\;mm/min$, depth 3 mm) requiring 6 s, processing efficiency is significantly enhanced, reducing per-hole processing time by approximately 37.2%. This not only substantially increases drilling speed but also ensures machining quality, achieving true synergistic optimization of “high quality” and “high efficiency” (compared with the experimental design in this paper). This optimization result not only validates the feasibility of the variable parameter process but also provides CFRP perforation parameters directly applicable to engineering practice, offering a practical solution to the delamination defect issues encountered in traditional processes.

### 3.4. Experimental Study on Variable Parameter Drilling of CFRP

**Figure 8:**
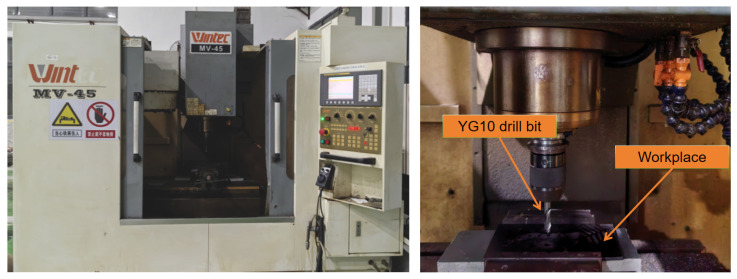
Schematic diagram of the experimental platform.

The experimental material is a unidirectional CFRP, with the model number being T700. The resin content is 60%. It is fabricated using the molding process. The layup form is unidirectional and there are a total of 20 layers. The experimental machine tool is the Daqiao Precision Machinery MV-45L vertical machining center, with a maximum spindle speed of 10,000 rpm. The drilling tool used is a YG10 drill bit with a diameter of 10 mm, a drill point angle of 130° and a helix angle of 30°. The schematic diagram of the drilling experimental platform is shown in Figure 8. In the processing parameters for drilling carbon fiber reinforced composite materials, the spindle speed is generally between 3000 and 24,000 rpm, and the feed rate is typically within the range of 25 to 100 mm/min [24].

**Figure 9:**
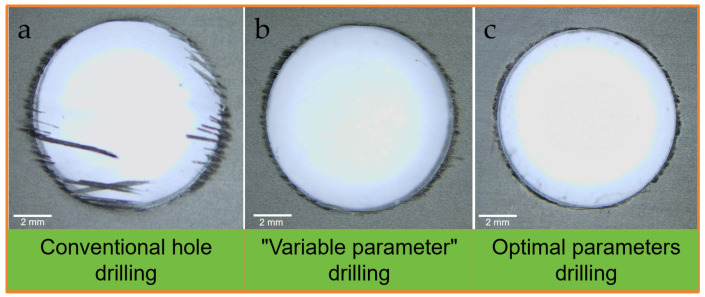
Processing quality of the drill hole exit of CFRP: (**a**) Conventional hole drilling; (**b**) “Variable parameter” drilling; (**c**) Optimal parameters drilling.

When drilling CFRP using different methods, all machining parameters remain consistent except for those at the drill exit point. Therefore, this paper focuses on the machining damage at the workpiece exit location. Each processing scheme underwent 20 replicate tests, with the test results aligning with simulation outcomes in terms of the changing trends of delamination damage. Figure 9 compares the exit quality of conventional drilling with “variable parameter” drilling, presenting representative damage morphologies from the three approaches. It is evident that all three machining methods exhibit varying degrees of delamination at the exit. Among them, conventional drilling produces the most severe burrs and delamination, while variable parameter drilling and its optimal parameter drilling scheme cause relatively milder machining damage.

## 4. Conclusions

1. A quantitative evaluation method for delamination damage factor was developed using the “MATLAB Sobel algorithm + ImageJ software” approach. This method solves the problem of insufficient accuracy in traditional qualitative observation and can effectively distinguish the delamination control effects of different process schemes (such as the conventional parameter $F_{A}$ = 1.690105 and the variable parameter $F_{A}$ = 1.49082). It provides a reliable basis for process evaluation.
2. The segmented variable-parameter drilling strategy of “high-to-low” has been proven effective in suppressing delamination damage at the exit. Experimental results show that compared with the traditional fixed-parameter process, this strategy reduces the stratification factor by 11.8%. Furthermore, under the conditions of this study, it is confirmed that the feed rate significantly influences delamination more than the spindle speed.
3. Based on genetic algorithm optimization, this study obtained a variable-parameter drilling process plan directly applicable to production guidance. This plan maximizes material removal rate while ensuring the delamination factor $F_{A}$≤ 1.6 (quality compliance), ultimately yielding optimal parameters (*n* = 3598 rpm, $f_{1}$= 132 mm/min, $f_{2}$= 92 mm/min). This provides validated, reliable parameters for efficient, high-quality drilling of CFRP components, offering direct practical value for manufacturing applications.

## Figures and Tables

**Table 1:** The damage factors corresponding to the four types of failure.

Damage Factor	Expression
$d_{ft}$ (Fiber tensile failure)	$d_{ft}=1-e^{-\sigma_{11}^{T}\varepsilon_{1t}^{f}L^{C}(\sqrt{F_{ft}}-1)/G_{ft}}/\sqrt{F_{ft}}$
$d_{fc}$ (Fiber compression failure)	$d_{fc}=1-e^{-\sigma_{11}^{C}\varepsilon_{1c}^{f}L^{C}(\sqrt{F_{fc}}-1)/G_{fc}}/\sqrt{F_{fc}}$
$d_{mt}$ (Failure by tensile stress along the vertical fiber direction)	$d_{mt}=1-e^{-\sigma_{22}^{T}\varepsilon_{2t}^{f}L^{C}(\sqrt{F_{mt}}-1)/G_{mt}}/\sqrt{F_{mt}}$
$d_{mc}$ (Compressive failure along the vertical fiber direction)	$d_{mc}=1-e^{-\sigma_{22}^{C}\varepsilon_{2c}^{f}L^{C}(\sqrt{F_{mc}}-1)/G_{mc}}/\sqrt{F_{mc}}$

**Table 2:** Cohesive unit material parameters.

*K_n_* (N/mm^2^)	*K_s_ = Kt* (N/mm^2^)	$\delta_{n}^{o}$ (MPa)	$\delta_{s}^{o}$ = $\delta_{t}^{o}$ (MPa)	*G_n_* (N/mm)	*G_s_ = G_t_* (N/mm)
4 × 10^6^	1 × 10^6^	60	90	0.2	1

**Table 3:** Tool material parameters.

Density	Elasticity Modulus	Poisson’s Ratio	Specific Heat	Heat Conductivity Coefficient
14,500 kg·m^−3^	640 GPa	0.22	220 J·(kg·°C)^−1^	75.4 W·(m·°C)^−1^

**Table 4:** CFRP material parameters.

		Room Temperature 298 K (25 °C)
Elastic modulus/GPa	*E*_11_	116
	*E*_22_	8.5
	*E*_33_	8.5
Poisson’s ratio	*V*_12_	0.02
	*V*_13_	0.02
	*V*_23_	0.28
Shear modulus/GPa	*G*_12_	3.26
	*G*_13_	3.26
	*G*_23_	2.12
Tensile strength/MPa	*X_T_*	1500
	*Y_T_*	27
Compressive strength/MPa	*X_C_*	900
	*Y_C_*	200
Shear strength/MPa	*S*_12_	80
	*S*_13_	80
	*S*_23_	80
Density/(kg·m^−3^)	*ρ*	1580

**Table 5:** Processing Scheme 1.

Spindle Speed (rpm)	Feed Rate (mm/min)
1000/2000/3000/4000/5000/6000	150

**Table 6:** Processing Scheme 2.

Spindle Speed (rpm)	Feed Rate (mm/min)
2000/4000	50/100/150

**Table 7:** Two processing schemes: conventional drilling and variable-parameter drilling.

	Machining Parameter	
Scheme 3 (Conventional Drilling)	*n* = 4000 rpm	*f* = 100 mm/min
Scheme 4 (Variable Feed Drilling)	*n*_1_ = 4000 rpm	*f*_1_ = 100 mm/min
	*n*_2_ = 4000 rpm	*f*_2_ = 50 mm/min

## Data Availability

The original contributions presented in the study are included in the article; further inquiries can be directed to the corresponding authors.
